# Elucidating distinct clinico-radiologic signatures in the borderland between neuromyelitis optica and multiple sclerosis

**DOI:** 10.1007/s00415-021-10619-1

**Published:** 2021-05-27

**Authors:** Maciej Juryńczyk, Elżbieta Klimiec-Moskal, Yazhuo Kong, Samuel Hurley, Silvia Messina, Tianrong Yeo, Mark Jenkinson, Maria Isabel Leite, Jacqueline Palace

**Affiliations:** 1 Department of Clinical Neurology, Nuffield Department of Clinical Neuroscienes, University of Oxford, Oxford, UK; 2 Laboratory of Brain Imaging, Neurobiology Center, Nencki Institute of Experimental Biology, Polish Academy of Sciences, Warsaw, Poland; 3 Department of Neurology, Jagiellonian University Medical College, Kraków, Poland; 4 Wellcome Centre for Integrative Neuroimaging, Nuffield Department of Clinical Neurosciences, University of Oxford, Oxford, UK; 5 CAS Key Laboratory of Behavioral Science, Institute of Psychology, Chinese Academy of Sciences, Beijing, 100101 China; 6 Department of Psychology, University of Chinese Academy of Sciences, Beijing, 100049 China

**Keywords:** Multiple sclerosis, Neuromyelitis optica, Optic neuritis, Myelitis, Magnetic resonance imaging, Prospective studies

## Abstract

**Background:**

Separating antibody-negative neuromyelitis optica spectrum disorders (NMOSD) from multiple sclerosis (MS) in borderline cases is extremely challenging due to lack of biomarkers. Elucidating different pathologies within the likely heterogenous antibody-negative NMOSD/MS overlap syndrome is, therefore, a major unmet need which would help avoid disability from inappropriate treatment.

**Objective:**

In this study we aimed to identify distinct subgroups within the antibody-negative NMOSD/MS overlap syndrome.

**Methods:**

Twenty-five relapsing antibody-negative patients with NMOSD features underwent a prospective brain and spinal cord MRI. Subgroups were identified by an unsupervised algorithm based on pre-selected NMOSD/MS discriminators.

**Results:**

Four subgroups were identified. Patients from Group 1 termed “MS-like” (*n* = 6) often had central vein sign and cortical lesions (83% and 67%, respectively). All patients from Group 2 (“spinal MS-like”, 8) had short-segment myelitis and no MS-like brain lesions. Group 3 (“classic NMO-like”, 6) had high percentage of bilateral optic neuritis and longitudinally extensive transverse myelitis (LETM, 80% and 60%, respectively) and normal brain appearance (100%). Group 4 (“NMO-like with brain involvement”, 5) typically had a history of NMOSD-like brain lesions and LETM. When compared with other groups, Group 4 had significantly decreased fractional anisotropy in non-lesioned tracts (0.46 vs. 0.49, *p* = 0.003) and decreased thalamus volume (0.84 vs. 0.98, *p* = 0.04).

**Conclusions:**

NMOSD/MS cohort contains distinct subgroups likely corresponding to different pathologies and requiring tailored treatment. We propose that non-conventional MRI might help optimise diagnosis in these challenging patients.

**Supplementary Information:**

The online version contains supplementary material available at 10.1007/s00415-021-10619-1.

## Introduction

Since the nineteenth century neuromyelitis optica (NMO, Devic’s syndrome) defined as acute simultaneous bilateral optic neuritis (BON) and transverse myelitis has been regarded as different from typical MS. The uniqueness of NMO has been emphasised by poor visual and motor outcome, and longitudinal extension of the transverse myelitis lesion (termed LETM) [1]. NMO was finally recognised as separate from MS when a subset of NMO patients were found to have serum antibodies against aquaporin-4 [2, 3]. This discovery broadened the spectrum of NMO (termed NMOSD) to include BON, isolated LETM and other limited forms of NMO [4].

Some patients with features of NMOSD have serum antibodies against MOG rather than aquaporin-4 [5, 6]. MOG-antibody disease (MOGAD) has similar brain imaging appearances to AQP4-antibody NMOSD, which are both easily distinguishable from MS [7]. In particular, AQP4-antibody NMOSD and MOGAD patients rarely fulfil MS brain lesion distribution criteria [8, 9].

The recognition of aquaporin-4-antibody NMOSD and MOGAD as separate diseases sparked interest in the cohort of patients with NMOSD features and red flags for the diagnosis of MS who are negative for both autoantibodies. It is widely accepted that this cohort is highly heterogenous and likely contains distinct diagnoses ranging from atypical multiple sclerosis to NMOSD mediated by yet undiscovered antibodies. Making ultimate diagnosis is very challenging as shown by high disagreement between experts when presented with individual cases [10]. According to the revised McDonald criteria for the diagnosis of MS, NMOSD should be considered in all patients with NMOSD features such as BON, severe brainstem involvement, longitudinally extensive spinal cord lesions, large cerebral lesions, or normal brain MRI [11]. It is well recognised that AQP4-antibody NMOSD and MOGAD patients can formally meet McDonald criteria [12–14]. A degree of diagnostic uncertainty in seronegative patients also occurs when applying NMOSD diagnostic criteria [15]. The correct diagnosis is however essential since typical NMOSD drugs are not licensed in MS nor are the treatments of choice, and MS-modifying drugs might exacerbate the course of NMOSD [16, 17].

In this study we analysed clinical, paraclinical and non-conventional imaging features of 25 antibody-negative patients recruited from the Specialist NMO Clinic in Oxford with recurrent syndromes at the borderline of MS and NMOSD, to identify patient subgroups, propose classification and guide clinicians when diagnosing and treating these challenging patients.

## Methods

### Patients

Patient inclusion criteria included (1) the presence of at least one NMOSD feature as per 2007 criteria [4], (2) seronegativity for both aquaporin-4 and MOG antibodies, (3) recurrent disease course.

### MRI scan acquisition

All participants underwent an MRI scan of the brain and cervical spinal cord in the Wellcome Centre for Integrative Neuroimaging in Oxford. The scan was obtained using a 3 T Siemens Prisma. The protocol sequences included T1 MP-RAGE, T2 FLAIR, T2 double inversion recovery (DIR), T2* mapping and diffusion tensor imaging (DTI) for the brain and T1 MP-RAGE and T2 FLAIR for the spinal cord.

### Image processing and analysis

The identification of cortical lesions on DIR was based on the consensus between two experienced raters (MJ, EKM). The presence or absence of the central vein sign was assessed according to the guidelines [18]. FLAIR images were scored for the presence of MS brain distribution criteria [8, 9]. Normalised brain volumes were obtained using FSL Sienax [19]. Subcortical structures and brainstem were segmented using FIRST and their volume was calculated using FSL command fslstats based on the label number of the structure of interest obtained from FIRST [20]. Cortical thickness was quantified using the Freesurfer software (version 6.0) [21]. To measure fractional anisotropy in non-lesioned tracts lesion masks were registered to T1, inverted and multiplied by masks of the white matter tracts of interest obtained from a DTI-based white matter atlas [22]. Freesurfer was implemented to obtain cortical ribbon masks, which were used when assessing mean diffusivity in the cortex. An R2*-weighted image was produced using a gradient echo MR sequence with a long echo time. A quantitative value for R2* was calculated in each voxel in units of [1/s].

The spinal cord was processed and automatically segmented using a deep learning algorithm implemented within the Spinal Cord Toolbox [23]. Vertebral disc labelling was performed manually and the cross-sectional area was calculated for each segment in the native space.

### Selection of discriminators for patient subgroup identification

Eleven NMO/MS discriminators were selected to separate patient subgroupings, including BON, poor residual visual acuity after optic neuritis (6/36 or worse in at least one eye), CSF oligoclonal bands unmatched in the serum, LETM, short-segment transverse myelitis, NMO-like brain lesions (all from clinical files or clinical scans performed prior to the study recruitment), MRI MS brain lesion distribution criteria (at least 1 lesion adjacent to the body of the lateral ventricle and in the inferior temporal lobe; or the presence of a subcortical U-fibre lesion; or a Dawson’s finger-type lesion) [8], cortical lesions, central vein sign, thalamus volume, fractional anisotropy in non-lesioned white matter tracts (as assessed on the prospective research images). Fractional anisotropy was measured in all non-lesioned tracts, apart from the optic radiation to exclude the potential effect of previous optic neuritis.

### Unsupervised identification of patient subgroups

**Fig. 1:**
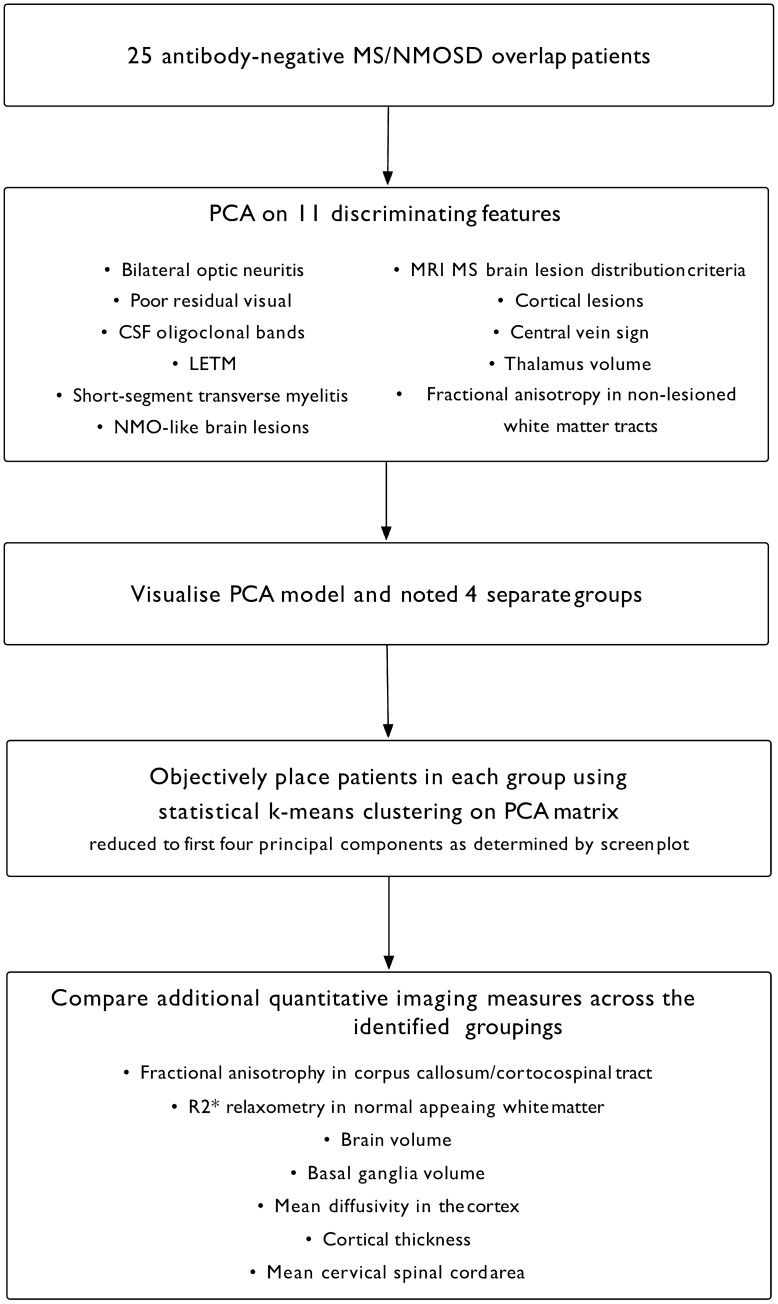
The flow diagram presents the sequence of analysis steps allowing for the unsupervised identification of antibody-negative NMOSD/multiple sclerosis patient subgroupings followed by exploration of tissue damage-related quantitaive imaging measures witin the identified groups

PCA on the obtained data matrix was performed using the prcomp function within the R statistical software. Visualisation of the first two principal components indicated the existence of four separate patient subgroups. To objectively determine which patients form each subgroup, *k*-means clustering (with four centres) was applied using *k*-means function from R software. As PCA normalises the data and uncorrelates the variables, the clustering was performed on the PCA matrix rather than original data. A scree plot was generated to show the proportion of variance explained by each principal component and to decide the number of principal components included in the matrix (the first four components, cumulatively explaining 69% of the variance, were ultimately used). Figure 1 shows the flow chart of the study.

### Quantitative and non-conventional imaging measures not selected as a priori discriminators

After subgroup identification various non-conventional imaging measures reflecting different aspects of tissue damage were analysed in the four identified subgroups: fractional anisotropy in corticospinal tracts, corpus callosum and optic radiation, R2* relaxometry in the normal-appearing white matter, normalised brain volume, normalised volume of basal ganglia (caudate, pallidum and putamen combined), mean diffusivity in the cortex, cortical thickness and mean cervical cord cross-sectional area). We selected fractional anisotropy rather than mean diffusivity as a measure of structural damage in the normal-appearing white matter [24, 25]. Mean diffusivity was used to assess damage in the cortex [26].

## Results

### Patients

Twenty-five patients were included in the study of whom 15 were female. Mean age at scan was 49 years (range 20–73) and median disease duration was 9.8 years (range 1–28). All patients were relapsing, their median number of attacks was three (range 2–11) and median EDMUS was three (range 0–8). Twelve patients had previous attacks of LETM, five had had BON and six were left with visual acuity at 6/36 or worse in at least one eye. Five patients were reported to have NMO-like brain lesions at some stage of their disease. Thirteen patients had CSF-exclusive oligoclonal bands. At the time of the research scan nine patients were not on any disease-modifying treatment, six were on azathioprine, five on methotrexate, two on mycophenolate mofetil, one on prednisolone alone, one on regular intravenous immunoglobulins and one on fingolimod. The breakdown of NMO- and MS-like features for individual patients is shown in Supplementary Table S1.

### Overview of brain lesions on the research scans

Eight out of 25 patients had distinct white matter lesions in the brain on FLAIR-weighted images (four had one lesion, one had two lesions, one had three lesions and two had four lesions). Five patients had Dawson’s finger(s), three had lesions adjacent to the body of lateral ventricle, two had infratentorial temporal lobe lesions and one had curved juxtacortical lesions. Seven patients fulfilled MS brain lesion distribution criteria [8]. Six had at least one lesion centred by a vein but only four fulfilled the ‘40% rule’ [27]. Five patients had at least one cortical lesion on FLAIR/DIR images.

### Patient subgroups

**Fig. 2:**
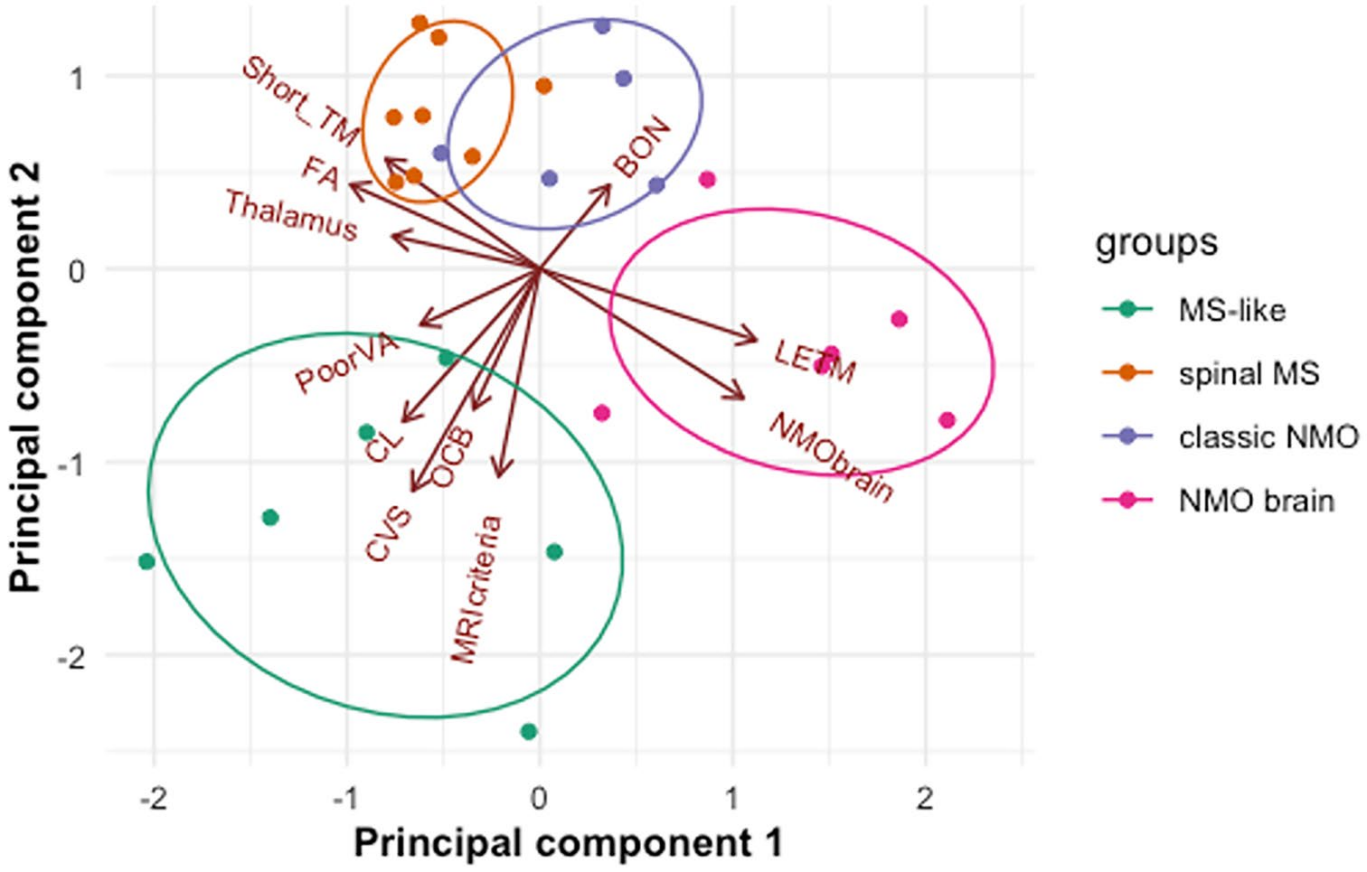
Principal component analysis plot shows localisation of each individual patient (represented as a dot) according to their scoring on two first principal components. Ellipses and label colours represent patient subgroupings as identified by *k*-means clustering with four centres. The plot is overlaid with eigenvectors showing how each discriminating feature contributes to the location of the patient on the graph. The clusters are named for convenience depending on their predominating clinical and imaging features. *BON* bilateral optic neuritis, *CL *cortical lesions, *CVS* central vein sign, *FA* fractional anisotropy in normal-appearing white matter tracts with the exclusion of optic radiation, *LETM* longitudinally extensive transverse myelitis, *MRIcriteria* MRI multiple sclerosis brain lesion distribution criteria, *NMObrain* neuromyelitis optica-like brain lesions, *OCB* oligoclonal bands in the cerebrospinal fluid unmatched for serum, *PoorVA* residual visual acuity at 6/36 or worse in at least one eye, *Short_TM* short-segment transverse myelits, *Thalamus* thalamus volume

Four separate subgroups were identified (Fig. 2). Group 1 was characterised by a high percentage of positivity for brain lesion distribution criteria (83%), central vein sign (83%) and cortical lesions (67%, Table 1). Group 2 consisted of patients who all had short-segment transverse myelitis but did not have MS brain lesions according to the criteria. Group 3 had a high percentage of BON and LETM (80% and 60%, respectively) but did not have any previous or current brain lesions (100%). Group 4 also consisted of LETM patients (100%) who, however, also typically had a history of NMOSD-like brain lesions (67%).

**Table 1:** Basic demographic, clinical information and breakdown of discriminating features in identified subgroups

	Group 1 (“MS”-like)	Group 2 (“Spinal MS”-like)	Group 3 (“Classic NMO”-like)	Group 4 (“NMO-like” with brain involvement)
Number of patients	6	8	5	6
Female %	17%	63%	80%	83%
Mean age at scan (years, range)	49 (21–73)	50 (41–64)	37 (20–58)	47 (24–70)
Median disease duration (years, range)	7 (2–19)	11 (4–28)	6 (1–13)	9 (2–20)
Mean EDMUS (range)	3 (0–7)	2.8 (0–5)	2.4 (1–5)	4 (2–8)
Bilateral ON	0%	0%	80%	17%
Poor visual acuity	33%	13%	40%	17%
CSF OCB	67%	50%	40%	50%
LETM	33%	13%	60%	100%
Short-segment TM	33.3%	100%	20%	17%
NMO-like brain lesions	17%	0%	0%	67%
MRI brain criteria	83%	0%	0%	33%
Cortical lesions	67%	13%	0%	0%
Central vein sign	83%	0%	0%	0%
FA	0.49 ± 0.01	0.49 ± 0.01	0.49 ± 0.01	0.46 ± 0.02**
Thalamus (cm^3^)	0.98 ± 0.13	0.97 ± 0.07	1.0 ± 0.05	0.84 ± 0.12*

These features were used to identify subgroups in the antibody-negative neuromyelitis optica/multiple sclerosis cohort using methods of unsupervised learning

The statistical significance of differences in non-conventional imaging measures across the subgroups is marked with asterisks: *p < 0.05, **p < 0.01

When compared with other groups, patients from Group 4 had significantly decreased fractional anisotropy in non-lesioned white matter tracts (0.46 ± 0.01 vs. 0.49 ± 0.01, *p* = 0.003) and decreased thalamus volume (0.84 ± 0.12 vs. 0.98 ± 0.08, *p* = 0.04). Table 1 shows basic demographic and clinical information on patients in each subgroup.

### Identified clusters correlate strongly with clinician’s diagnosis

Comparison with clinician’s diagnosis revealed that likely MS was diagnosed only in patients from Group 1 and Group 2 (83% and 88%, respectively, Table 2), while likely NMOSD was diagnosed only in Group 3 and 4 (80% and 83%, respectively). Taking into account the breakdown of discriminating features and diagnoses for convenience we have termed Group 1 “MS-like”, Group 2 “spinal MS-like”, Group 3 “classic NMO”-like and Group 4 “NMO-like with brain involvement”.

**Table 2:** Comparison between subgroups identified by unsupervised machine learning and clinician’s diagnosis

	Group 1 (“MS”-like)	Group 2 (“Spinal MS”-like)	Group 3 (“Classic NMO”-like)	Group 4 (“NMO-like” with brain involvement)
Number of patients	6	8	5	6
MS diagnosis	83%	88%	0%	0%
NMO diagnosis	0%	0%	80%	83%
Other/undetermined	17%	12%	20%	17%

### Quantitative imaging differences between the identified groups in tissue damage parameters not used for subgroup identification

Table 3 shows non-conventional imaging differences between four identified subgroups in parameters representing various aspects of disease pathology: normal-appearing white matter damage (fractional anisotropy in distinct white matter tracts, R2* relaxometry), axonal damage (normalised brain and subcortical structure volumes), cortical damage (mean diffusivity in the cortex, cortical thickness) and spinal cord damage (mean cervical spinal cord area).

**Table 3:** Non-conventional magnetic resonance imaging measures in identified subgroups

	Group 1 (“MS”-like)	Group 2 (“Spinal MS”-like)	Group 3 (“Classic NMO”-like)	Group 4 (“NMO-like” with brain involvement)
Fractional anisotropy in corpus callosum	0.56 ± 0.02	0.58 ± 0.02	0.59 ± 0.02	0.48 ± 0.04***
Fractional anisotropy in corticospinal tracts	0.44 ± 0.02	0.44 ± 0.01	0.43 ± 0.01	0.43 ± 0.01
Fractional anisotropy in optic radiation	0.52 ± 0.03	0.55 ± 0.02	0.54 ± 0.01	0.51 ± 0.05
Mean R2* relaxometry in the normal-appearing white matter	21.2 ± 0.58	20.9 ± 1.0	21.5 ± 1.0	20.8 ± 0.7
Mean R2* relaxometry in the basal ganglia	29.5 ± 5.1	28.9 ± 2.9	28 ± 2.8	27.9 ± 4.2
Normalised brain volume (l)	1.48 ± 0.14	1.48 ± 0.1	1.50 ± 0.09	1.36 ± 0.08
Normalised basal ganglia volume (cm^3^)	13.3 ± 1.6	13.4 ± 1.2	12.3 ± 1.6	11.4 ± 2.2
Mean diffusivity in the cortex	0.87 ± 0.02	0.87 ± 0.03	0.86 ± 0.04	0.92 ± 0.03*
Mean cortical thickness	2.74 ± 0.1	2.70 ± 0.07	2.77 ± 0.13	2.66 ± 0.06
Mean cervical spinal cord area	61.4 ± 4.3	57.7 ± 6.8	65.7 ± 5.1	53.1 ± 6.5*

These measures were not used for subgroup identification

Statisitcally significant differences are marked with stars in the last column

*p < 0.05

**p < 0.01

***p < 0.001

### Group 1 and Group 2

**Fig. 3:**
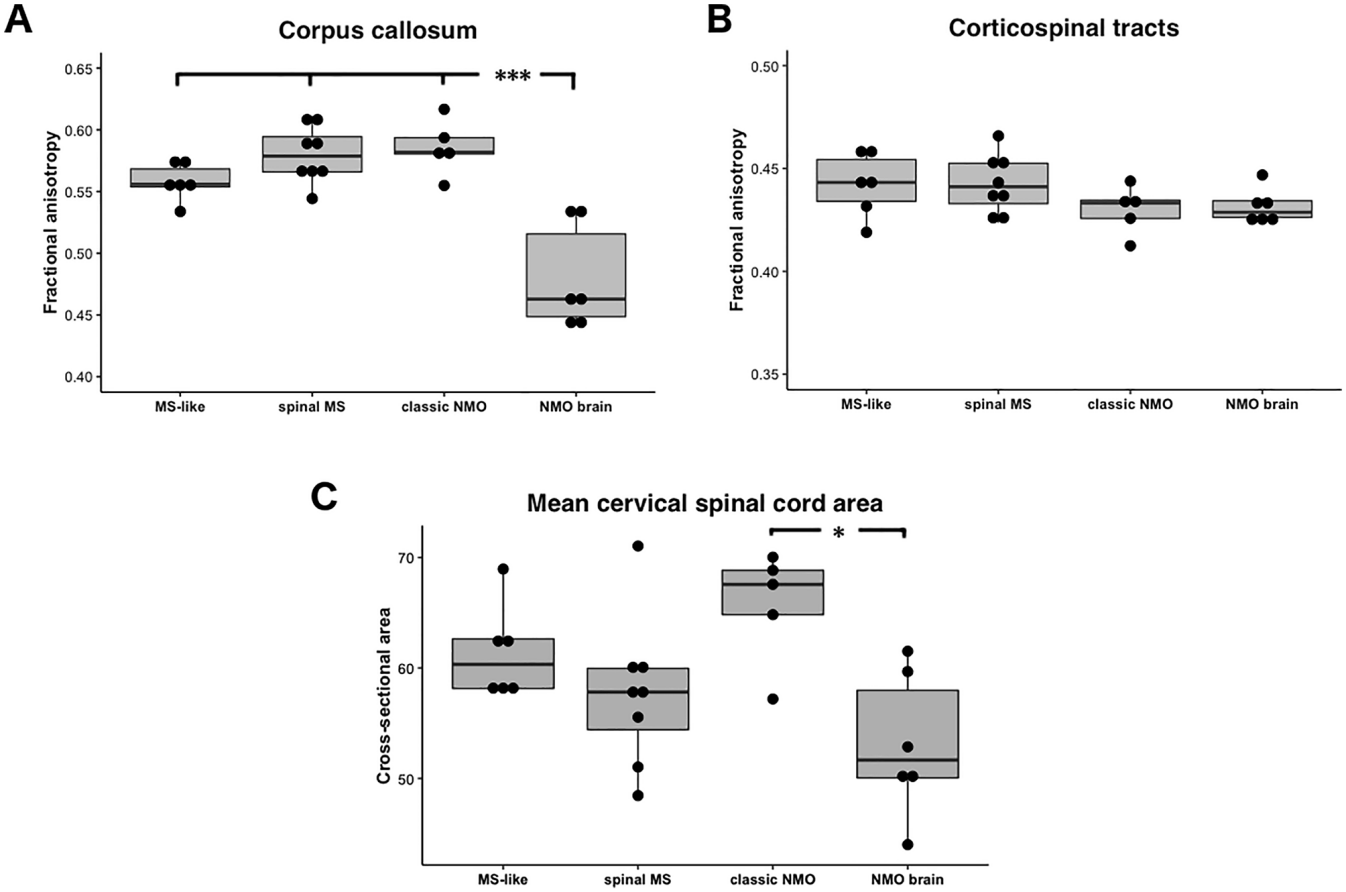
Fractional anisotropy in the corpus callosum (**A**) and corticospinal tracts (**B**) in each identified subgroup. Group 4 shows significantly lower fractional anisotropy in the corpus callosum as compared with other groups (***p < 0.001), but no between-group difference is observed in corticospinal tracts. (**C**) Mean spinal cord cross-sectional area averaged across all eight cervical segments in four identified subgroups. Group 4 had significantly more atrophy than Group 3 (*p = 0.01). Statistically significant differences are marked with asterisks

**Fig. 4:**
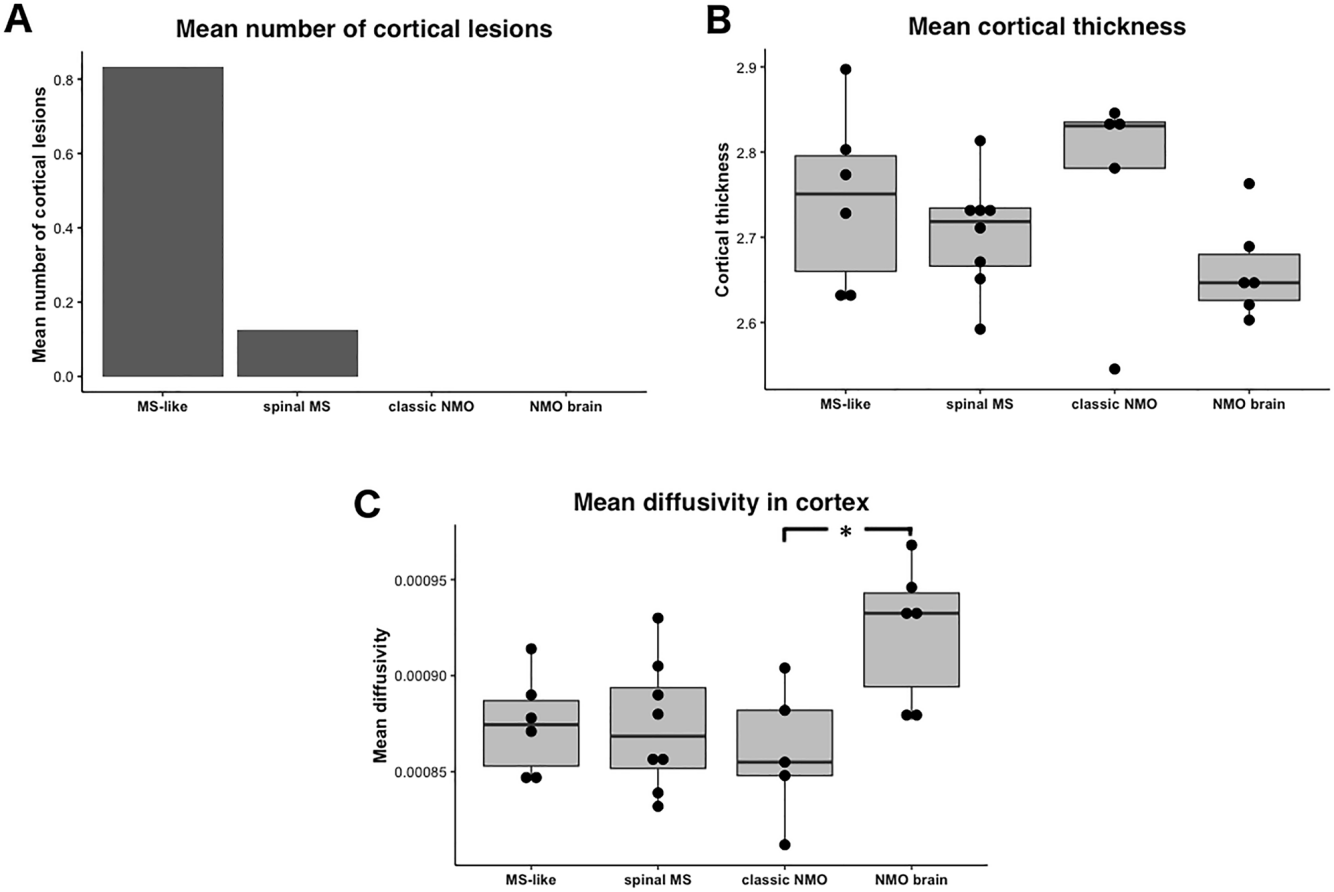
Different aspects of cortical pathology are shown across four groups (**A**) mean number of cortical lesions, (**B**) mean cortical thickness, (**C**) mean diffusivity in the cortex. Mean diffusivity in the cortex was significantly higher in Group 4 as compared with Group 3 (*p = 0.02, marked with an asterisk)

‘MS-like’ and ‘spinal MS-like’ patients did not differ significantly between each other in terms of normal-appearing white matter tract integrity (Fig. 3A, 3B) or atrophy measures in the brain but the latter group had a lower mean cross-sectional area in the cervical spinal cord (57.7 ± 6.8 vs. 61.4 ± 4.3, non-significant, Fig. 3C). Both groups combined also had a trend for higher R2* values in the thalamus compared to patients from NMO-like groups (21.9 ± 1.2 vs. 20.9 ± 1.0, Table 3). Only patients from the MS-like groups had cortical lesions (Fig. 4A) and, as expected, these were more common in patients with white matter brain lesions (Group 1) than in patients with predominantly spinal MS-like disease (Group 2).

### Group 3

Despite high proportion of previous LETM history (60%) “classic NMO-like” patients did not appear to have spinal cord atrophy and had significantly higher mean cervical spinal cord cross-sectional area when compared to “NMO-like with brain involvement” patients (65.7 ± 5.1 vs. 53.1 ± 6.5, *p* = 0.01, Fig. 3B). “Classic NMO-like” patients also had high cortical thickness and absence of cortical lesions, which all suggested absence of cortical pathology in this group (Fig. 4).

### Group 4

Given that low fractional anisotropy in normal-appearing white matter tracts (with the exclusion of the optic radiation) strongly contributed to the generation of “NMO-like with brain involvement” subgroup we analysed whether this difference is attributed to any particular white matter tract. Interestingly, we have found significant differences in fractional anisotropy in the corpus callosum when comparing between Group 4 and each of the three other groups (*p* < 0.001, Tukey’s test), but not in corticospinal tracts (Fig. 3, Table 3). Cerebral volumetric measures (total brain, basal ganglia, brainstem volume, cortical thickness) were generally lower in this group as compared to other groups but this was not statistically significant (Table 3). Importantly, patients from this group had significantly higher mean diffusivity in the cortex as compared with “classic NMO-like” and “spinal MS-like” patients (Fig. 4).

## Discussion

We present the results of a prospective cross-sectional study of 25 double antibody-negative relapsing patients with NMOSD features and uncertain diagnosis. The aim of the study was to group the patients in an unsupervised, unbiased way into pathologically and/or clinically distinct subgroups on the basis of MS/NMOSD discriminatory features. We have found four separate groups existing in the cohort – two with predominantly MS-like features – in the brain (Group 1) or in the spinal cord (Group 2), one with optico-spinal “classic NMO-like” presentation (Group 3) and one with predominantly spinal cord and brain NMOSD-like disease (Group 4). We examined differences in non-conventional imaging findings. which were not used for subgroup identification, and found that while there were no significant differences between Groups 1 and 2, Group 4 displayed significantly more damage in the brain and spinal cord as compared with Group 3.

Current knowledge of antibody-negative NMOSD and the borderland between MS and NMOSD is very limited, as this group of patients has been largely neglected from previous studies, most likely due to difficulties in the analysis resulting from diagnostic uncertainty. It is understood that this group might include atypical MS, NMOSD mediated by yet undiscovered antibodies and other conditions such as neurosarcoid. The condition can be disabling in its own right and more so if treated inappropriately [28]. Most expert clinicians would opt for global immunosuppression rather than MS-modifying drugs in case of diagnostic doubt [10] but it is unclear how effective this approach is in terms of preventing disability. Interestingly, antibody-negative NMOSD appears to respond differently to treatment as compared with aquaporin-4-antibody-positive NMOSD. In recent clinical trials neither satralizumab, anti-IL-6 antibody, nor inebilizumab, anti-CD19 drug, showed any signal of efficacy in antibody-negative NMOSD [29–31]. The latter finding was particularly interesting as B cell-depleting therapy is often considered by clinicians to be the most appropriate for patients with overlapping symptoms of NMOSD and MS. All this points to a compelling need to classify the antibody-negative cases to help choose appropriate treatment.

There are several limitations of the study. These include the supervised choice of discriminators, which might bias the generation of clusters. To reduce this bias we included only the most typical NMOSD and MS features (see “Methods”) combined with well-established or promising non-conventional imaging discriminators (cortical lesions [32], central vein sign [33], thalamus volume, fractional anisotropy in normal-appearing white matter [34]). In the analysis multiple imaging outcomes were used for a small number of patients however most non-conventional measures were assessed after the subgroups have been identified to explore if there might be differences in the underlying tissue damage patterns/pathologies between the groups. The small number of patients is due to the relative rarity of antibody-negative NMOSD patients [35] and their frequent labelling as atypical MS. Of note, we focussed only on relapsing patients who definitely have a chronic disease and excluded monophasic illness, for example viral or post-viral, which typically would not be considered for long-term disease-modifying treatment. Although rare, relapsing antibody-negative NMOSD patients cause the most difficulty in the clinic in terms of diagnosis and thus treatment decisions [10]. Another weakness is the lack of a gold standard reference for the ultimate diagnosis, which is also the reason to do this study and why the literature in this area is sparse.

Patients diagnosed with likely antibody-negative NMO in the clinic were all assigned to Group 3 or 4 by the unsupervised algorithm, and these groups differed significantly between each other. Patients from Group 4 had highly destructive disease of the spinal cord and brain as seen in advanced MS, however, they all had a number of red flags for the diagnosis of MS as well. First, all these patients had a history of LETM, considered a hallmark of NMOSD [36] and extremely rare in MS [37]. Four out of six had NMO-like brain lesions, including a lesion adjacent to the 3rd ventricle (patient 3), periaqueductal and hypothalamic lesions (patient 13), oedematous lesion involving the complete thickness of the splenium of corpus callosum (patient 20) and a large hemispheric white matter lesion (patient 21). One patient in this group had a dramatic exacerbation on natalizumab with a formation of ring-enhancing brain lesions (patient 12). These patients are likely to have a disease process that is different from MS, potentially mediated by autoantibodies or other type of inflammation. One of these patients underwent biopsy of the cerebral lesion (Patient 21) which showed chronic inflammation and reactive gliosis without evidence of MS demyelination, granulomatous process, vasculitis or neoplasm. This group may represent a new disease entity.

All patients from Group 3 had a normal brain appearance based on standard imaging and significantly less brain atrophy or white matter disintegrity when compared with Group 4. These patients had predominantly optico-spinal presentation but they had less spinal cord atrophy than Group 4 despite a high proportion of previous LETM in both groups. Two out of five had attacks of both BON and LETM, two had recurrent isolated optic neuritis including at least one attack of simultaneous BON and one had LETM followed several months later by unilateral optic neuritis. Two out of five in this group were left with poor residual visual acuity. This group, as opposed to other groups, did not have any evidence of cortical damage, was the least disabled of all groups (average EDMUS 2.4) and appeared to have a milder form of NMOSD with a high proportion of BON, similar to what is observed in MOGAD [13]. We find it likely that disease process in this group is mediated by yet undiscovered antibodies.

Patients from Groups 1 and 2, despite being referred to NMO clinic, appear to have forms of MS. Brain lesions in these patients, although there were typically only a few of them, were positive for landmark MS features (Group 1). Those without brain lesions (Group 2) had short-segment lesions in the spinal cord but very rarely had NMOSD features, such as LETM (12.5%), BON (0%), poor visual recovery (12.5%) or NMO brain lesions (0%). Of the patients in this group, 50% had unmatched oligoclonal bands in the CSF. This form of spinal MS may turn out to be pathologically distinct and is worth further research.

In conclusion, to our knowledge this is the first study focussing on the heterogenous cohort of antibody-negative NMO/MS patients using non-conventional imaging and unsupervised unbiased clustering algorithms to subclassify these patients and guide treatment decisions. Our results suggest that differential diagnosis in NMOSD/MS patients might benefit from the use of novel imaging techniques not yet widely used in clinical practice, including central vein sign, cortical lesions and the implementation of MS MRI brain lesion distribution criteria. If present these biomarkers might direct clinicians towards the likely diagnosis of MS. We suspect patients who are negative for these features and have a normal brain imaging might still have the diagnosis of MS if they present with spinal short-segment disease either isolated or associated with unilateral optic neuritis. We also believe that patients with a history of LETM have a non-MS disease. Those with LETM who have a history of BON might have NMOSD mediated by yet undiscovered antibodies, while those with MS-atypical brain lesions and significant brain atrophy could possibly have a diagnosis alternative to MS or ‘true’ NMOSD. Further prospective studies in identified subgroups, correlated with clinico-pathological studies and antibody research, will help clarify the underlying processes in each group.

## Supplementary Information

Below is the link to the electronic supplementary material.

Supplementary file1 (DOCX 16 KB)

## Data Availability

All data and material are available from the corresponding authors upon request.

## Abbreviations

BON: Bilateral optic neuritis
DIR: Double inversion recovery
DTI: Diffusion tensor imaging
FLAIR: Fluid attenuated inversion recovery
LETM: Longitudinally extensive transverse myelitis
MOG: Myelin oligodendrocyte glycoprotein
NMOSD: Neuromyelitis optica spectrum disorders
PCA: Principal component analysis

## Code availability

Custom codes are available from the corresponding authors upon request.
