# Supplementary material for: Elucidating distinct clinico-radiologic signatures in the borderland between neuromyelitis optica and multiple sclerosis
Source: J Neurol. 2021 May 27;269(1):269–79. doi: 10.1007/s00415-021-10619-1 (PMC8738499; doi:10.1007/s00415-021-10619-1)
Supplement: Supplementary file 1 — Supplementary file1 (DOCX 16 KB) [file 415_2021_10619_MOESM1_ESM.docx]

**Supplementary Table S1**

| Patient code | LETM | Bilateral optic neuritis | Recurrent optic neuritis | Poor visual acuity | NMO-like brain lesions | Absence of MS-like brain lesions | Absence of CSF-exlcusive bands |
| --- | --- | --- | --- | --- | --- | --- | --- |
| 02 |  |  |  |  |  | X |  |
| 03 | X |  |  |  | X | X |  |
| 05 |  |  |  | X |  |  |  |
| 06 |  |  |  |  |  | X | X |
| 07 |  |  |  |  |  | X | X |
| 08 | X |  |  |  |  | X | X |
| 09 | X | X |  |  |  | X | X |
| 10 |  |  | X | X |  | X |  |
| 11 |  | X | X | X |  | X |  |
| 12 | X |  | X | X |  | X |  |
| 13 | X |  |  |  | X | X | X |
| 14 |  |  |  |  |  | X |  |
| 15 | X |  |  |  |  |  |  |
| 16 | X |  |  |  | X | X |  |
| 17 |  |  | X |  |  | X |  |
| 18 |  |  |  |  |  | X |  |
| 19 | x |  |  |  |  | x | X |
| 20 | X | X |  |  | X |  | X |
| 21 | X |  |  |  | X | X |  |
| 22 |  | X | X |  |  | X | X |
| 24 | X | X | X |  |  | X |  |
| 25 |  |  |  |  |  | X | X |
| 26 |  |  | X |  |  | X | X |
| 27 | X |  |  | X |  | X | X |
| 28 |  |  | X | X |  |  |  |
